# Genome-Wide DNA Methylation in Policemen Working in Cities Differing by Major Sources of Air Pollution

**DOI:** 10.3390/ijms23031666

**Published:** 2022-01-31

**Authors:** Katerina Honkova, Andrea Rossnerova, Irena Chvojkova, Alena Milcova, Hasmik Margaryan, Anna Pastorkova, Antonin Ambroz, Pavel Rossner, Vitezslav Jirik, Jiri Rubes, Radim J. Sram, Jan Topinka

**Affiliations:** 1 Department of Genetic Toxicology and Epigenetics, Institute of Experimental Medicine CAS, Videnska 1083, 142 20 Prague 4, Czech Republic; andrea.rossnerova@iem.cas.cz (A.R.); irena.chvojkova@iem.cas.cz (I.C.); alena.milcova@iem.cas.cz (A.M.); hasmik.margaryan@iem.cas.cz (H.M.); radim.sram@iem.cas.cz (R.J.S.); jan.topinka@iem.cas.cz (J.T.); 2 Department of Nanotoxicology and Molecular Epidemiology, Institute of Experimental Medicine CAS, Videnska 1083, 142 20 Prague 4, Czech Republic; anna.pastorkova@iem.cas.cz (A.P.); antonin.ambroz@iem.cas.cz (A.A.); pavel.rossner@iem.cas.cz (P.R.J.); 3 Centre for Epidemiological Research, Faculty of Medicine, University of Ostrava, Syllabova 19, 703 00 Ostrava, Czech Republic; vitezslav.jirik@osu.cz; 4 Veterinary Research Institute, Hudcova 296/70, 621 00 Brno, Czech Republic; rubes@vri.cz

**Keywords:** air pollution, DNA methylation, environment, molecular epidemiology, epigenetics

## Abstract

DNA methylation is the most studied epigenetic mechanism that regulates gene expression, and it can serve as a useful biomarker of prior environmental exposure and future health outcomes. This study focused on DNA methylation profiles in a human cohort, comprising 125 nonsmoking city policemen (sampled twice), living and working in three localities (Prague, Ostrava and Ceske Budejovice) of the Czech Republic, who spent the majority of their working time outdoors. The main characterization of the localities, differing by major sources of air pollution, was defined by the stationary air pollution monitoring of PM2.5, B[a]P and NO_2_. DNA methylation was analyzed by a genome-wide microarray method. No season-specific DNA methylation pattern was discovered; however, we identified 13,643 differentially methylated CpG loci (DML) for a comparison between the Prague and Ostrava groups. The most significant DML was cg10123377 (log_2_FC = −1.92, *p* = 8.30 × 10^−4^) and loci annotated to *RPTOR* (total 20 CpG loci). We also found two hypomethylated loci annotated to the DNA repair gene *XRCC5.* Groups of DML annotated to the same gene were linked to diabetes mellitus (*KCNQ1*), respiratory diseases (*PTPRN2*), the dopaminergic system of the brain and neurodegenerative diseases (*NR4A2*). The most significant possibly affected pathway was Axon guidance, with 86 potentially deregulated genes near DML. The cluster of gene sets that could be affected by DNA methylation in the Ostrava groups mainly includes the neuronal functions and biological processes of cell junctions and adhesion assembly. The study demonstrates that the differences in the type of air pollution between localities can affect a unique change in DNA methylation profiles across the human genome.

## 1. Introduction

Long-term exposure to air pollutants has an adverse health impact and affects the genome and epigenome [1]. DNA methylation is one of the most studied epigenetic mechanisms that regulates gene expression and affects genome stability [2]. DNA methylation can serve as a useful biomarker of exposure, and an analysis of DNA methylation gives a better understanding of the effects of environmental exposure, as well as the role of epigenetic mechanisms, on human health [3,4]. CpG loci, as significant biomarkers of exposure, were summarized in a recent review [5]; they were further identified as a biomarker of tobacco smoke exposure in adults [6] and in smoking pregnant women [7,8], and were linked with alcohol consumption [9]. CpG loci were also found as environmental biomarkers for cumulative exposure to lead [10].

There is a wide range of methodological approaches for measuring the level of DNA methylation. A commonly used method is quantitative global DNA methylation, which represents an effective, fast and cheap solution [11,12]. However, this method cannot distinguish between important hypo- and hypermethylated sites. Other methods are used in epigenome-wide association studies (EWAS) [13]; these are currently mostly based on gene-specific genome-wide DNA methylation (microarray approaches).

The effect of air pollution was widely studied in children, who are more sensitive than adults, and in newborns, to clarify the effect of prenatal exposure [14,15]. In these studies, the authors found an association between prenatal exposure to NO_2_ and particulate matter (PM) and significant CpG loci that regulate activity of genes associated with asthma and other pulmonary diseases. DNA methylation alterations induced by benzo(a)pyrene (B[a]P) contributed partially to abnormal DNA methylation in air pollution-related lung cancer samples. These changes may affect the development and progression of lung cancer [16].

Czech studies focused on the impact of air pollution on the human genome have a long tradition [17]; a contributory factor is that one of the air pollution hot-spots in Europe is situated in the Moravian-Silesian district, in particular in the Ostrava region. In Ostrava, the highest concentrations of B[a]P and fine PM < 2.5 µm (PM2.5) recorded in the Czech Republic are consistently being detected [18]. Thus, inhabitants of this locality have often been studied in molecular epidemiological research [19]. DNA methylation profiles were examined in asthmatic and healthy children [20]. Whole-genome gene expression was assessed in newborns from districts with different levels of air pollution [21], and city policemen [22,23] who worked in cities with different concentrations of air pollutants. The assessment of cancerogenic risk after the ambient air inhalation in inhabitants of industrial and non-industrial localities was also performed [24]. In the city of Ostrava, depending on the locality, the prevalence of respiratory diseases, such as asthma, or recurrent airway inflammation, is higher than the national average [25,26,27].

This study is a continuation of a long-term research project on air pollution’s impact on the human genome. In this part, using microarray EPIC BeadChips (Illumina), we evaluated the genome-wide gene-specific DNA methylation in city policemen from three localities in the Czech Republic: Ostrava, Prague and Ceske Budejovice (CB). The study can be regarded as both an occupational and environmental exposure investigation because the city policemen were living in the study localities and, simultaneously, they spent most of their working time outdoors, where they were mostly exposed to engine emissions from car traffic, and to pollutants from local industry. Here, we aimed to find specific DNA methylation patterns based on the quantitative data of the annotated 856,865 CpG sites, which could be characterized by work and life in environments affected by different types of air pollution.

## 2. Results

### 2.1. Characteristics of Study Subjects

The data on 125 city policemen (250 samples) were obtained from detailed questionnaires describing personal information, exposure history and lifestyle factors. All subjects were nonsmoking males, who had been residents in the study cities for at least 3 years. Table 1 shows the most important data, characterizing the potential differences between the groups. The age spectrum of policemen covered the entire productive age, ranging from 21 to 63 years. No differences (ANOVA, *p* = 0.61) in age between the city groups were observed (40.4 vs. 39.4 vs. 38.0, respectively). The BMI of subjects covered normal weight to obese with similar distributions in all localities. Mean values were almost identical for all study localities (28.6 vs. 28.4 vs. 28.2, respectively) and without statistical significance (ANOVA, *p* = 0.85). In the last row of Table 1, the occupation duration is shown. Most of the subjects had a long occupation history as city policemen, with no significant differences between localities (ANOVA, *p* = 0.13; mean values are 13.94 vs. 11.78 vs. 10.22, respectively). Only six subjects in Prague, four subjects in Ostrava and four subjects in CB, have worked as policemen for less than 2 years and they have all completed at least secondary education. The health data in the questionnaires showed that all policemen were of normal health without specific dietary issues or excessive alcohol consumption.

### 2.2. Air Pollution Monitoring

The concentrations of air pollutants (Table 2A), used for the characterization of the air pollution in the study cities in the three-month period before sample collection, were obtained from the Czech Hydrometeorological Institute (CHMI; Annual tabular overview). Further details regarding measuring frequencies and stations are described in Section 4.2.

In Ostrava, the concentrations of PM2.5 were the highest during both seasons compared to other localities (*p* < 0.05 for Ostrava vs. others). Interestingly, the concentrations of PM2.5 in Prague and CB were comparable (*p* = 0.42 and *p* = 0.15 for winter and summer, respectively).

The concentrations of B[a]P did not significantly differ between Ostrava and Prague in winter (*p* = 0.29). However, it was not the mean but rather the median in the Ostrava and Prague winter that showed a greater difference (2.6 vs. 1.6). We found a significant difference between Ostrava and Prague in summer (*p* < 0.05), and also between Ostrava and CB (*p* < 0.05 for both seasons).

There were no differences in the NO_2_ concentrations in winter between Ostrava and other localities (*p* = 0.38 and *p* = 0.05 for Prague and CB, respectively). However, during the summer season, significant differences were found in all localities (*p* < 0.05 for both Ostrava vs. Prague and Ostrava vs. CB). The highest NO_2_ concentration was observed in Prague in summer.

For a broader comparison of long-term exposure, we summarized the mean annual concentrations of these air pollutants in the last four years (Table 2B).

The concentrations of pollutants were relatively stable over the years. In 2019, the lowest levels of air pollution were noted in all cities compared to previous years. The differences between the localities correspond with the data shown in Table 2A. The highest concentrations of NO_2_ were detected in Prague when compared with Ostrava.

### 2.3. General DNA Methylation Profiling

**Figure A1:**
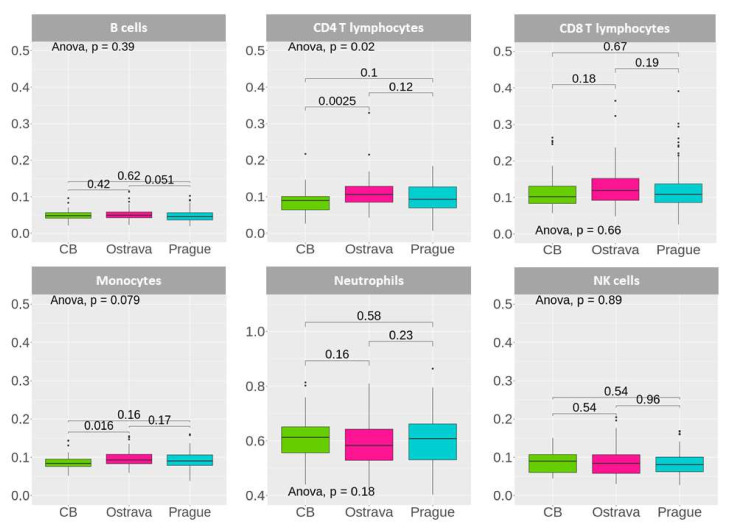
T lymphocytes—Th helper cells; CD8 T lymphocytes—Tc cytotoxic cells; NK cells—natural killer cells (between CB, Ostrava and Prague samples).

First, we analyzed the proportion of blood cell types in samples grouped to localities to exclude the possible effects of sample collection. The results are shown in Figure A1 (Appendix A). The proportion of B cells, Tc cytotoxic cells (CD8T), neutrophils and natural killer cells had a stable, comparable profile in all groups. The results for the proportion of monocytes were on the border of significance. We found a significant variance (*p* < 0.05) in Th helper cells (CD4T) between Ostrava and CB. Due to the low number of samples from CB, we decided not to correct this discrepancy.

**Figure 1:**
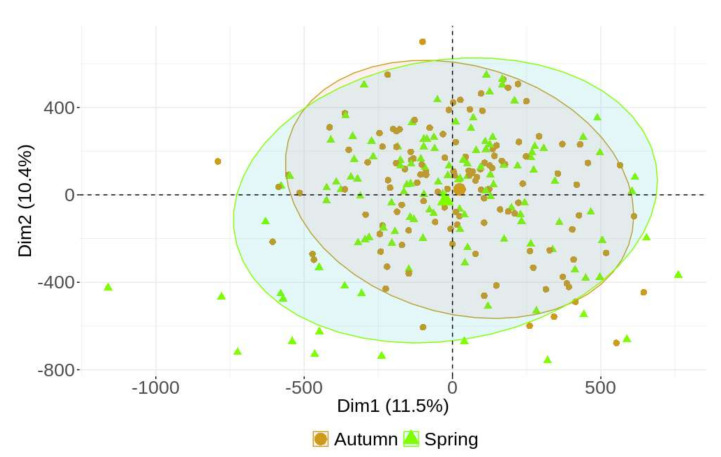
PCA shows no differences between the spring and autumn sampling periods.

Before comparing DNA methylation profiles between the three localities (Ostrava, Prague and CB), we verified the effect of season (regardless of locality, N = 125 for each season) on the differences in DNA methylation. We did not observe any season-specific clustering of DNA methylation profiles, nor did we detect any differentially methylated CpG loci (DML) (Figure 1).

We used all 250 samples from both seasons for the comparison of DNA methylation profiles between localities. Based on the results of air pollution monitoring, Ostrava was considered as a polluted locality, while Prague and CB were used as control regions, although the localities have specific air quality profiles given by various sources of air pollution (Table 2A,B). The comparison was set on (a) Ostrava–Prague; (b) Ostrava–CB and (c) Prague–CB.

**Figure 2:**
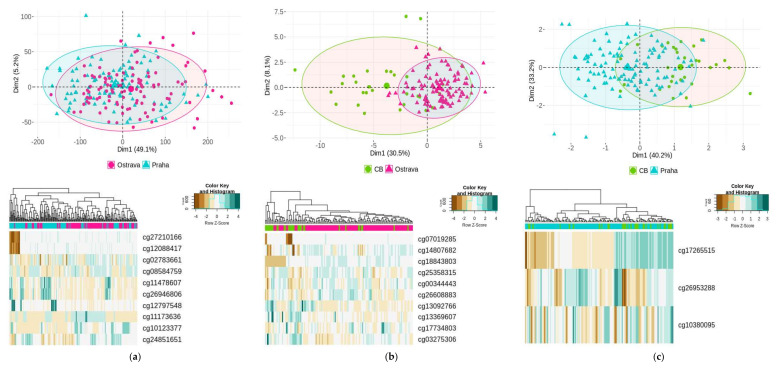
General DNA methylation profiling represented by PCA and heatmaps for comparison: (**a**) Ostrava–Prague; (**b**) Ostrava–CB; (**c**) Prague–CB. In the heatmap, hierarchical clustering for all samples (represented by columns) for the most significant DML are shown (n = 10, n = 10, n = 3, respectively).

The results of the DNA methylation pattern clustering, analyzed by PCA, and the cluster heatmap applied on significant DML for all compared groups, are shown in Figure 2.

A total of 13,643 CpG loci (FDR, *p* < 0.01) were significantly differentially methylated (8859 hypermethylated and 4784 hypomethylated) when samples of (a) Ostrava–Prague were compared. A total of 31 CpG loci (FDR, *p* < 0.01) differed in samples of (b) Ostrava–CB (22 hypermethylated and 9 hypomethylated), while only 3 DML (FDR, *p* < 0.01) were found in samples of (c) Prague–CB (1 hypermethylated and 2 hypomethylated).

The CpG loci shown in Figure 2 were summarized in a basic overview, consisting of their relation to CpG islands (open sea, island, N/S shore and N/S shelf), a localization of the chromosome, a reference gene, and a level of significance (presented in Table 3). From these loci, two (cg12088417 and cg27210166) for group (a) were hypomethylated, potentially affecting *RPTOR,* a gene encoding the regulatory-associated protein of MTOR complex 1. Strongly hypermethylated CpG locus (cg18843803, log_2_FC = 1.95), which can affect the protein-coding gene *TSHZ3* (teashirt zinc finger homeobox 3), was identified in group (b). Only three differentially methylated loci were found in group (c). Strong hypomethylation of cg17265515 (log_2_FC = −1.59) can regulate the gene *ERICH1* (glutamate-rich 1). All DML are listed in Table S1.

We mostly found a different proportion of DML in CpG islands in group (b) compared to all evaluated CpG. In group (a), there were more Open Sea loci than in all evaluated CpG.

**Figure 3:**
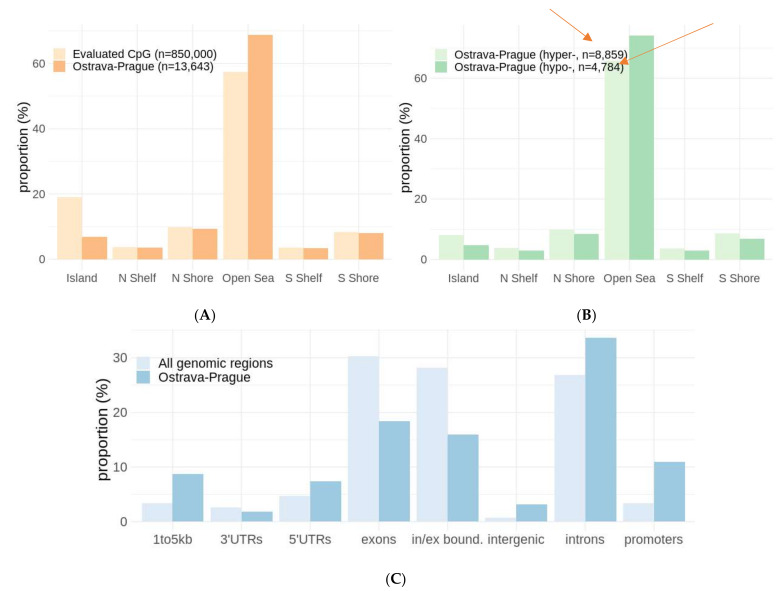
A proportion of annotated CpG loci according to distance from CpG islands for differentially methylated sites for the Ostrava–Prague group compared to all evaluated CpG loci (**A**), where the highest representation of loci is in Open Sea. A resolution to hypomethylated and hypermethylated sites is comparable (**B**). A proportion of annotated methylated genomic regions to gene position shows a higher proportion of promoters in Ostrava–Prague annotated genomic regions than in all genomic regions (**C**).

In group (a), in which we could annotate methylation regions, the location of DML was characterized in relation to putative promoter regions, 3′UTR, exons, introns, etc. We detected a three-times-higher frequency of DNA methylation in the promoter regions than in all annotated genomic regions (Figure 3C). All proportions are shown in Figure 3A–C.

### 2.4. Differentially Methylated Groups of Loci and Geneset Enrichment

For the Ostrava–Prague comparison, we detected differentially methylated groups (DMG) with numerous DML. DMG with more than seven CpG are listed in Table S2. Some of those located in Island/Shore regions with high relevance to health impacts are shown in Table 4A and discussed in Section 3. For further analysis, we selected groups with more than 15 CpG sites (Table 4B). A total of 20 CpG sites were annotated to the *RPTOR* gene, which is also shown in Table 3, with the most differentially hypomethylated loci (cg12088417, cg27210166). The *COL23A1* encodes a transmembrane nonfibrillar collagen. It is potentially regulated by 17 DML, of which 15 are hypermethylated. We also found the 16 CpG loci annotated to the gene that plays a key role in cardiac action potential (*KCNQ1). PTPRN2,* annotated to 16 mostly hypomethylated DML, encodes the tyrosine phosphatase, a major islet autoantigen in type-1 diabetes.

**Figure 4:**
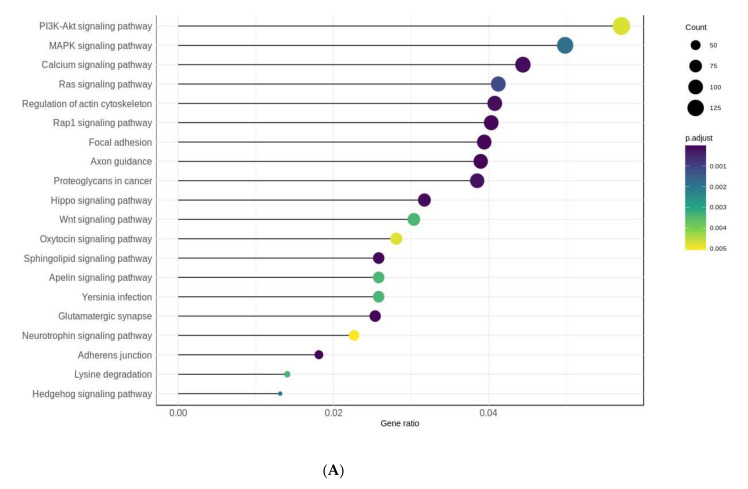

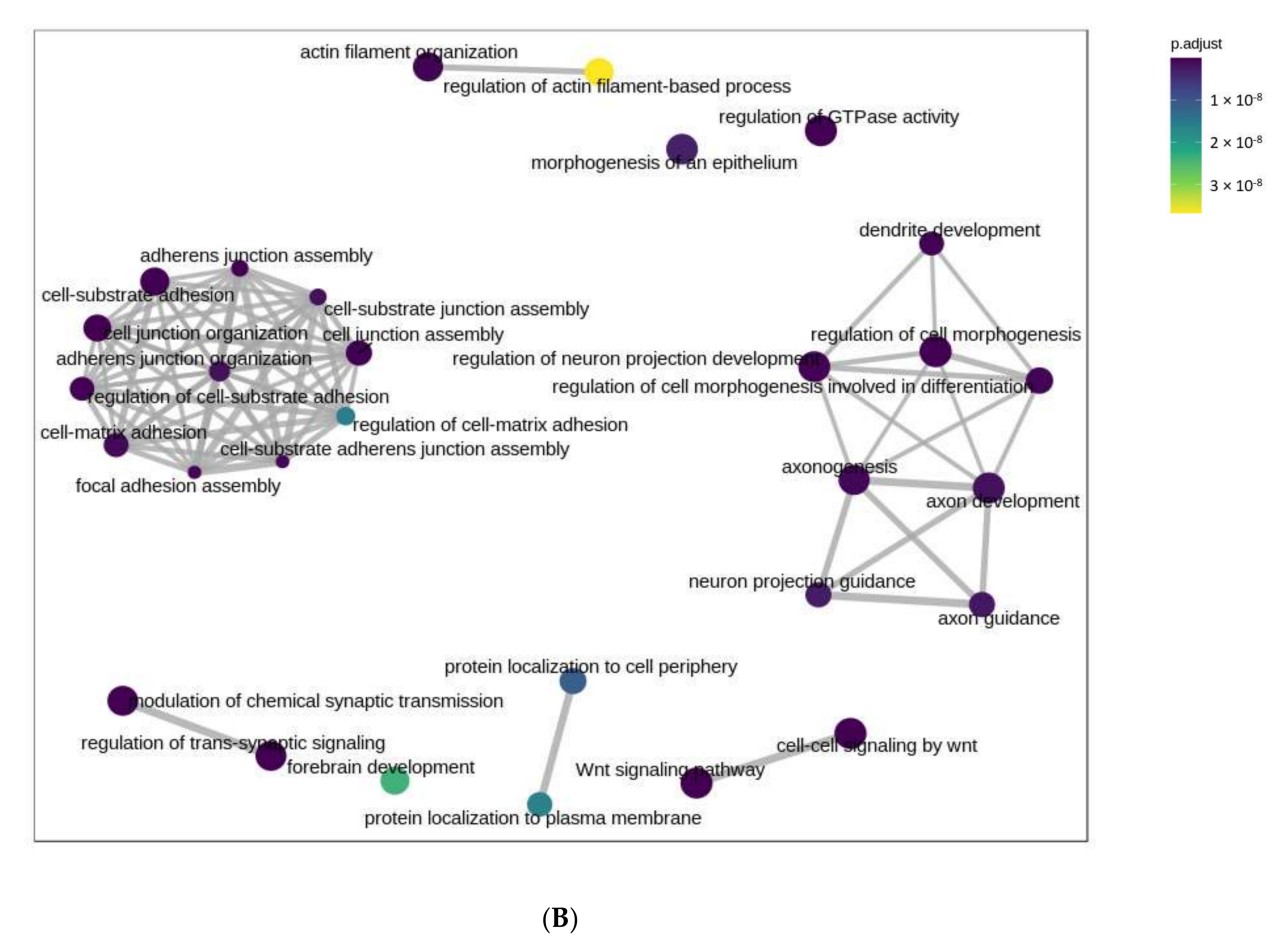
Gene ontology characterized by KEGG-affected pathways for genes annotated to significant CpG loci (**A**). The gene set enrichment map based on the distance of each gene in biological processes (**B**).

For 13,643 CpG loci in the Ostrava–Prague comparison, 5881 genes were annotated (based on ETREZID). These genes were used for gene ontology and gene set enrichment (Figure 4A,B). We obtained significant Kyoto Encyclopedia of Genes and Genomes (KEGG) pathways with more than 50 potentially regulated genes in pathways (Figure 4A), among which signaling cascades prevail. The significant potentially affected pathways (adj. *p*-value < 0.01) with the highest number of genes were the PIK3A-Akt signaling pathway (hsa04151, 126 genes) and the MAPK signaling pathway (hsa04010, 110 genes). The most significant pathway (adj. *p*-value = 1.45 × 10^−6^) was Axon guidance (hsa04360, 86 genes). The genes usually formed an overlap of several biological pathways, creating higher functional units—biological processes. Functional clusters (Figure 4B) based on measuring distances between the genes were formed mainly for adherens junction assembly and neuronal functions and development, which covered more than 100 regulated genes in each individual process. Other significant pathways (adj. *p*-value < 0.05) are summarized in Table S3.

## 3. Discussion

We report the unique study of gene-specific genome-wide DNA methylation profiles in 125 policemen working in cities in the Czech Republic with various types of air pollution. The assessment of the effect of air pollution is not straightforward; therefore, we tried to reduce the impact of lifestyle factors as much as possible by selecting similar cohorts (Table 1). The study is part of a complex project, “Healthy Aging in an Industrial Environment”, the main aim of which is to find specific biochemical, genetic and epigenetic biomarkers of effect, mainly caused by air pollution exposure in different localities and in various population groups. In policemen, we sampled the same cohort in two rounds (in spring and in autumn, at the end of the winter and summer seasons, respectively). The experimental design, based on the analysis of different seasons in Ostrava policemen, was used in a recent study of semen quality and sperm DNA integrity [30]. The authors showed that sperm chromatin damage and the percentage of immature sperm were highly sensitive to air pollution. We also assessed the effect of seasons on DNA methylation patterns, but as no effect and no differentially methylated loci were detected (Figure 1), we included all samples of the policemen (N = 250) in the analysis. We hypothesize that the short-term effects of different levels of air pollution in individual seasons do not influence the DNA methylation pattern. This assumption is based on the fact that there are periods in human life when DNA methylation profiles are susceptible to change (prenatal, early childhood and older age) [31], while DNA methylation is generally relatively stable. On the other hand, another study described the effects of a 24-h short-term exposure to PM2.5 on genome-wide DNA methylation, using the same platform (Illumina Infinium Human Methylation EPIC BeadChip) that we applied in our study [4]. However, the authors used controlled indoor exposure for a short time, and they detected only a small number of DML.

For the characterization of localities and impact of air pollution on DNA methylation patterns, we compared three pollutants in the three-month period before both sampling points, as in a previous study [20]. Moreover, we used annual mean concentrations for four consecutive years. In Ostrava, the highest levels of PM2.5 and B[a]P were detected, while PM2.5 concentrations were comparable in Prague and CB. No differences in NO_2_ concentrations in winter were observed between Ostrava and the other two localities. The highest long term annual mean concentrations of NO_2_ were identified in Prague, where the traffic intensity and NO_2_ levels along/near roads showed positive correlation [32]. The authors of this study used passive samplers at 65 locations in Prague, and found that 32% of locations exceeded the EU annual limit of 40 µg/m^3^.

We concluded that the localities were mostly characterized by diverse types of air pollution, rather than by high differences in the concentration of air pollutants. The main cause of air pollution in Prague is road traffic, where intensities exceed all other cities in the Czech Republic. Ostrava is characterized by common winter inversions; the city is located close to the Polish border with heavy industry and coal mines producing high levels of air pollutants, along with local industrial emissions. However, air quality in the city has shown an improving trend over the last decades [18].

By comparing the proportion of blood cell types, we found a significant variance in CD4T between the Ostrava and CB samples (Appendix A). We hypothesize that the discrepancies between the groups may be related to low sample numbers in CB. On the other hand, the differences in cell proportion can also be an important regulatory mechanism in response to the environment [33,34].

After merging the sampling periods and through the analysis of 250 samples, we found 13,643 CpG loci for the Ostrava–Prague comparison. Only 31 DML were detected for the Ostrava and CB comparison; we further observed three DML when the Prague and CB samples were compared. We detected almost twice the hypermethylated DML than hypomethylated ones, and a three-times-higher frequency of promoter methylation than in all the annotated genomic regions. The loci in promoters are more biologically significant with the potential to affect gene expression [35].

Our previous study was focused on the whole-genome gene expression response to long-term exposure to air pollution in men from Ostrava and Prague during several seasons [22]. Although no clear relationship between concentrations of air pollutants and gene expression profiles was found, the reduced gene expression was observed in the Ostrava men compared to the controls. In the Ostrava cohort, an increased expression of *XRCC*5 was found [23]. *XRCC5* encodes the protein Ku80, a key player of the DNA repair mechanism NHEJ, in which it binds the DNA breaks [36]. In this study, we found the two hypomethylated loci (cg23433242 and cg01633232; Table S1) annotated to *XRCC5*. Regardless of whether we found differential DNA methylation in other NHEJ genes as in [23], we can hypothesize that epigenetic modification of a single gene might be sufficient to affect DNA repair functions [37].

Comparing Ostrava and Prague, the most hypomethylated DML was found to be CpG locus cg10123377. This CpG locus was also found in the study of patients with systematic lupus erythematosus in CD8T+ cells [38]. Two of the hypermethylated loci (cg11478607 and cg26946806) located in Island and S Shore, respectively, are annotated to the *GSTT1*-encoding xenobiotic metabolizing enzyme, which plays an important role in human carcinogenesis [39] and impacts the markers of genotoxicity [40]. Single-nucleotide polymorphisms (SNPs) in *GSST1* were widely studied in relation to the interaction effects of long-term air pollution exposure on the risk of acute myocardial infarction and hypertension, with high susceptibility to air pollution being a promoter of coronary vulnerability [41].

Comparing Ostrava and CB, we found the strongly hypermethylated CpG locus (cg18843803), which can affect the protein-coding gene *TSHZ3*. This gene controls the development of diverse components of the circuitry required for breathing. The protein Teashirt 3 regulates the development of neurons involved in both the respiratory rhythm and airflow control [42]. The reduced expression of this gene and the consequent caspase upregulation may be correlated with the progression of Alzheimer’s disease [43].

In more detail, we focused on groups of differentially methylated loci annotated to the same gene in Ostrava samples, compared to the Prague samples (above seven CpG in Table S2; and a selection of genes with the highest proportion of groups of DML in Table 4A,B).

The highest number of groups of DML was annotated to the *RPTOR* gene (N = 20). RPTOR is a key component in mTOR pathway, a cell-signaling pathway commonly deregulated in human cancer, which plays roles in mRNA translation, autophagy, cell growth and immune responses (it restricts proinflammatory and promotes an anti-inflammatory response) [44,45]. RPTOR responds to nutrient and insulin levels to regulate cell growth [46]. The deregulation of this gene and pathway is associated with various human diseases, including cancer and diabetes [47,48]. The mTOR pathway (hsa04150) was also found among the significantly affected KEGG pathways (Table S3) that contain 59 genes which are potentially regulated by DML.

DMG, with the second highest number of DML, regulates *COL23A1*. Collagen XXIII is considered as a biomarker for the detection and recurrence of non-small-cell lung carcinoma and the reappearance of prostate cancer [49,50]. A total of 16 CpG loci, mostly hypermethylated, are annotated to the *KCNQ1* gene, which encodes the voltage-gated potassium channel required for the repolarization phase of the cardiac action potential [51]. A hypermethylation of *KCNQ1* is associated with poor semen parameters or male infertility [52]. Another study showed, by identifying temporal differences in the imprinting status and methylation effects, that the intronic *KCNQ1* locus mediates susceptibility to type-2 diabetes [53]. A major autoantigen associated with insulin-dependent diabetes mellitus [54], *PTPRN2* (protein tyrosine phosphatase), was identified for the 16 mostly hypomethylated CpG loci. Methylation in *PTPRN2* is associated with childhood asthma and chronical obstruction pulmonary disease in adulthood [55], and it is also related to residential proximity to major roadways in the placenta samples of pregnant women [56].

Nine hypermethylated CpG loci in the Shore/Island region regulate *NR4A2*, a member of the nuclear receptor family of intracellular transcription factors (adopted from GeneCards). *NR4A2* plays a key role in the maintenance of the dopaminergic system of the brain [57], and it is also highly expressed in peripheral blood leukocytes [58]. *NR4A2* is involved in autoimmune and neurodegenerative diseases, especially in Parkinson’s disease, where the reduced expression of this gene in peripheral blood can be considered as a potential biomarker for diagnosis, and a promising approach to therapy [59,60,61,62]. Another nine hypermethylated CpG sites can reduce the expression of *CDK2AP1,* which encodes cyclin-dependent kinase 2 (CDK2), which is important in the reducing of cell proliferation, contributes to cell cycle termination, and is a known tumor suppressor [63].

The most significantly affected pathway, the Axon guidance (hsa04360), contains 86 potentially regulated genes. Changes in the expression or function of these proteins might induce pathological changes in neural circuits that predispose to, or cause, neurological diseases [64]. Several of the genes in this pathway, which were found close to demethylated loci, were identified in patients with early-onset Alzheimer’s disease [65]. The largest cluster presented in Figure 4B gathered the most significant biological processes, with considerable potential gene regulation in neuronal functions. The second firm cluster includes the functions of cell junctions and adhesion assembly. These biological functions are potentially affected by DNA methylation in the Ostrava sample groups compared to Prague.

This study is first that involved a large set of policemen from different air-polluted cities, in whom genome-wide gene-specific DNA methylation was evaluated. It is well known that DNA methylation is a useful biomarker of prior environmental exposure and future health outcomes. However, it can be also affected by many factors of lifestyle and lifetime exposure. Although we could not determine all these factors and separate a specific role of air pollution, we identified the potential biomarkers that will be further studied. For a follow-up study, we have extensive questionnaire data of the complete exposure history of every individual, which will be linked with DNA methylation results to obtain more comprehensive outcomes in this molecular-epidemiological study.

## 4. Materials and Methods

### 4.1. Study Subjects

The study subjects were 125 male city policemen working in three cities in the Czech Republic (Prague, N = 55; Ostrava, N = 54; CB, N = 16), all involved in the project in two rounds in 2019: spring (March/April) and autumn (September/October). The cohorts of policemen were chosen as groups that spend most of their working time outdoors. Different cities are characterized, among other factors, by different levels and types of air pollution. Prague is the capital of the Czech Republic and its most densely populated area. Road traffic most significantly contributes to the level of air pollution in Prague. Ostrava is the third largest city, with a long history of coal mining and heavy industry. To date, the Ostrava region is known as one of the European hot spots of air pollution due to its geographical location close to the industrial region of Poland, frequent inversions in winter, heavy traffic and local industrial emissions. The third city, CB, was usually selected as a control locality in our previous studies [21,66,67], due to its close proximity to the Sumava National Park and the large agricultural area in the district (more details given in Table 1).

### 4.2. Air Pollution Monitoring

Daily concentrations of selected air pollutants (PM2.5, B[a]P and NO_2_) during both three-month periods before sampling (winter and summer), as well as the annual averages from 2016 to 2019, were obtained from the Annual tabular overview, CHMI (http://portal.chmi.cz/?l=en (accessed on 27 January 2022)). Data were acquired by automatic air pollution monitoring in each city. The stations were located representatively for patrol activities of city policemen. In the city of Ostrava, we used the main CHMI station in Ostrava–Poruba located in a residential area close to a gas station. In Prague 5, we selected two stations: for B[a]P and NO_2_ monitoring, a station in Prague–Reporyje was situated in a school garden; for PM2.5, a station was placed in Prague–Stodulky in a housing estate. In CB, the stations were located in urban and residential areas. The measurement frequency was daily in the case of PM2.5 and NO_2_. For B[a]P, the data were obtained twice a week.

### 4.3. DNA Methylation Analysis

A total of 250 samples of the venous blood of 125 policemen (every policeman provided samples in two sampling periods) were collected into vacuettes containing ethylenediaminetetraacetic acid (EDTA), and frozen at −20 °C for later use. Genomic DNA (gDNA) was extracted using Miller’s salting out method [68]. gDNA (1000 ng) was treated overnight with sodium bisulfite using the EZ DNA Methylation Kit (Zymo Research, Irvine, CA, USA) for the conversion of unmethylated cytosines to uracils, while methylated cytosines remained unchanged. The bisulfite-converted DNA (BCD) samples were stored at −20 °C until use. BCD was processed using the Infinium Methylation EPIC Kit (Illumina, San Diego, CA, USA) according to the manufacturer’s protocol (Infinium HD Methylation Assay Protocol) including enzymatic fragmentation, precipitation and hybridization, followed by BeadChip washing and staining. Each BeadChip consisted of 8 samples. The chips allowed the detection of over 850,000 methylation sites per sample across the genome at single-nucleotide resolution. The methylation status at each CpG site, scanned by the iScan System (Illumina, San Diego, CA, USA), was estimated by measuring the intensity of the pair of methylated and unmethylated probes.

### 4.4. Statistical Analysis

The descriptive statistics of epidemiological data from questionnaires (age, BMI, exposure history), air pollution (differences in concentrations) and cell type proportion, were carried out using R-stats. Depending on the distribution of the data, the t-test or the nonparametric Mann–Whitney Sum U-test was used for the comparison of individual groups and ANOVA was used for factor analysis. All advanced statistical analyses related to methylation were processed using scripting in an R environment.

Raw microarray data were downloaded as *idat* files, imported to the R environment and processed with the minfi package [69]. Data were normalized using the quantile method. A series of filtering was performed. Probes with SNPs at CpG sites and the cross-reactive probes were also excluded to obtain the resulting number of 794,441 probes [70]. We estimated associations between principal components and slide factors, and used the Combat function (sva package) for batch correction [71].

Beta values for the determination of the level of methylation as the ratio of the fluorescent signals from the methylated vs. unmethylated sites were also calculated using the minfi package. Preprocessing analyses were performed to study the distribution of beta values and the variation of methylation across all samples.

PCA was performed to identify the variance using covariance matrix, and to detect the potential effects of season and locality. We identified differentially methylated loci using the top Table function (limma package) [72]. For multiple testing of the false discovery rate (FDR), the *p*-values for the contrast of interest were adjusted to be below <0.01, which is regarded to be the most appropriate for microarray analysis [73].

The proportions of genomic regions to gene positions were analyzed using the annotatr package [74]. An annotation of the CpG site to ENTREZID, plots of the KEGG pathways and an enrichment map were obtained using clusterProfiler package v4.0 [75]. The proportions of blood cell types presented in Appendix A were calculated using the ENmix package [76].

## 5. Conclusions

This study focused on the comparison of DNA methylation profiles in city policemen working and living in localities that differ in terms of major sources of air pollution. The sampling was conducted repeatedly in two seasons (spring and autumn in the same year). The obtained results clearly demonstrated that there was no effect of season, which corresponds with relatively slow changes in DNA methylation settings in adults. On the other hand, the effects of the different localities with various exposure profiles were clearly visible. For genetic toxicology, the results indicating differences in xenobiotics metabolism and repair and neurodevelopment pathways can be considered the most significant. However, the associations of DNA methylation changes with various diseases, particularly diabetes mellitus or neurodegenerative or respiratory diseases, are often reported. Our observations support the hypothesis that epigenome modification is an effective process for the optimal management of the genome function in response to various types of environments, but it is also a risk factor for future disease development.

## Figures and Tables

**Table 1:** Basic characteristics of subjects grouped according to the study locality.

Study Locality	Ostrava	Prague	CB
Number of samples/subjects	108/54	110/55	32/16
Age (years)	40.4; 9.3 (42.0; 21–61)	39.3; 9.2 (38.0; 23–63)	38.0; 6.4 (38.0; 22–48)
Mean; SD (median; min–max)			
BMI	28.6; 4.1 (28.3; 20.4–44.8)	28.4; 3.9 (28.7; 19.4–36.8)	28.2; 3.7 (27.3; 23.2–41.0)
Mean; SD (median; min–max)			
Occupation duration (years)	13.9; 7.9 (14.0; 0.8–26.8)	11.8; 6.7 (12.0; 1.1–25.9)	10.2; 7.5 (10.0; 0.8–26.5)
Mean; SD (median; min–max)			

**Table 2:** (**A**) Concentrations of selected air pollutants in study cities during the three-month period before sampling. (**B**) A comparison of annual mean concentrations of PM2.5, B[a]P and NO_2_ in all study cities in 2016–2019.

(A)							
		Ostrava		Prague		CB	
		Winter	Summer	Winter	Summer	Winter	Summer
**PM2.5**	Mean; SD	24.4; 19.1	11.3; 5.3	15.0; 13.4	9.1; 4.2	15.4; 11.9	9.7; 4.2
µg/m^3^	Median (min–max)	19.8 (2.6–101.8)	10.3 (3.6–40.5)	10.4 (1.5–57.4)	8.3 (2.0–22.2)	12.3 (1.5–47.6)	9.1 (3.3–22.2)
**B[a]P**	Mean; SD	4.1; 3.3	0.3; 0.3	3.6; 3.0	0.1; 0.1	2.00; 1.01	0.1; 0.1
ng/m^3^	Median (min–max)	2.6 (0.3–13.5)	0.1 (0.0–1.3)	1.6 (0.0–16.7)	0.0 (0.0–0.5)	1.7 (0.60–5.10)	0.0 (0.0–0.9)
**NO_2_**	Mean; SD	19.9; 9.4	10.9; 3.70	19.5; 8.9	17.9; 5.5	17.6; 8.2	9.8; 2.6
µg/m^3^	Median (min–max)	19.0 (4.5–51.5)	10.3 (4.4–23.2)	16.9 (4.7–47.9)	18.3 (8.3–32.9)	14.1 (4.8–44.4)	9.8 (3.4–18.3)
(**B**)							
**PM2.5 (µg/m^3^)**				2016	2017	2018	2019
Ostrava		Mean; SD		22.2; 18.3	21.7; 15.9	22.9; 18.2	17.4; 14.4
Prague				16.5; 13.8	16.7; 12.2.	18.0; 15.2	12.3; 9.9
CB				18.5; 14.8	14.6; 10.4	16.0; 13.6	12.8; 10.4
**B[a]P (ng/m^3^)**				2016	2017	2018	2019
Ostrava		Mean; SD		2.2; 0.9	2.5; 0.7	2.9; 0.9	2.0; 0.6
Prague *				0.8; 0.3	0.9; 0.3	0.8; 0.2	0.7; 0.2
CB				1.5; 0.5	1.3; 0.5	1.1; 0.3	1.2; 0.4
**NO_2_ (µg/m^3^)**				2016	2017	2018	2019
Ostrava		Mean; SD		16.4; 15.0	16.2; 13.6	17.2; 15.4	15.2; 13.5
Prague ^#^				25.6; 24.0	31.0; 29.1	33.0; 31.8	28.6; 27.5
CB				15.7; 14.2	15.4; 13.7	14.9; 13.8	12.9; 6.9

*# Due to incomplete data from the Prague 5 station, we used information from other stations in other districts: Prague 4 (B[a]P)—a 50 m distance from a main road; Prague 1 (NO_2_)—the main square in the city center.

**Table 3:** Description of the 10 (or 3) most DML in samples from (**a**) Ostrava–Prague, (**b**) Ostrava–CB and (**c**) Prague–CB.

	CpG Locus	Chromosome	Relation to Island *	Gene	log_2_FC	Adj *p*-Value
**(a)** **Ostrava–Prague**	cg10123377	3	Open Sea	*--*	−1.92	8.30 × 10^−4^
	cg24851651	11	S Shelf	*CCS*	−1.21	8.53 × 10^−3^
	cg11173636	10	Open Sea	*RP11-170M17.1*	−1.19	4.04 × 10^−3^
	cg27210166	17	Open Sea	*RPTOR*	−0.90	2.55 × 10^−3^
	cg08584759	10	Island	*C10orf47*	−0.87	8.30 × 10^−4^
	cg12088417	17	Open Sea	*RPTOR*	−0.86	7.05 × 10^−4^
	cg02783661	12	S Shelf	*CCDC77*	−0.78	2.91 × 10^−3^
	cg11478607	22	Island	*GSTT1*	0.76	8.62 × 10^−3^
	cg26946806	22	S Shore	*GSTT1*	0.81	8.37 × 10^−3^
	cg12797548	1	Open Sea	*NME7*	0.83	6.69 × 10^−4^
**(b)** **Ostrava–CB**	cg13092766	2	Island	*BCL2L11*	−0.91	7.49 × 10^−3^
	cg13369607	19	Island	*SAFB2*	−0.63	5.44 × 10^−3^
	cg03275306	15	Open Sea	*BCL2A1*	−0.53	3.63 × 10^−3^
	cg00344443	11	Island	*AP000797.3*	0.53	7.49 × 10^−3^
	cg25358315	2	Island	*CAPG*	0.57	3.63 × 10^−3^
	cg14807682	8	Island	--	0.57	7.49 × 10^−3^
	cg17734803	1	Island	*FOXD3-AS1*	0.62	7.49 × 10^−3^
	cg26608883	11	Island	*CALCB*	0.71	7.49 × 10^−3^
	cg07019285	10	Open Sea	*CALML3-AS1*	0.87	9.86 × 10^−3^
	cg18843803	19	Open Sea	*TSHZ3*	1.95	3.63 × 10^−3^
**(c)** **Prague–CB**	cg17265515	8	Open Sea	*ERICH1*	−1.59	5.05 × 10^−3^
	cg10380095	7	Open Sea	--	−0.31	5.05 × 10^−3^
	cg26953288	8	S Shore	*BAI1*	0.55	1.48 × 10^−3^

Abbreviations: *CCS* (copper chaperone for superoxide dismutase), *RP11-170M17.1* (NA), *RPTOR* (regulatory-associated protein of MTOR complex 1), *C10orf47* (also known as *PROSER2*, proline- and serine-rich 2), *CCDC77* (coiled-coil domain containing 77), *GSTT1* (glutathione S-transferase theta 1), *NME7* (NME/NM23 family member 7), *BCL2L11* (BCL2-like 11), *SAFB2* (scaffold attachment factor B2), *BCL2A1* (bcl-2-related protein A1), *AP000797.3* (NA), *CAPG* (capping actin protein), *FOXD3-AS1* (FOXD3 antisense RNA 1), *CALCB* (calcitonin-related polypeptide beta), *CALML3-AS1* (CALML3 antisense RNA 1), *TSHZ3* (teashirt zinc finger homeobox 3), *ERICH1* (glutamate-rich 1), *BAI1* (adhesion G protein-coupled receptor B1); --- (out of gene).* Gene transcription is dependent on location of the CpG loci in the genome [28]. Islands are densely covered by CpG sites, mostly situated in promoters of housekeeping genes. Shores are tissue-specific regions with lower density of CpG sites, 2 kb distance from CpG islands. Shelves (2–4 kb from CpG island) and Open sea (>4 kb from CpG island) are more dynamic regions [29].

**Table 4:** (**A**) Selected DMG with high relevance to health impacts discussed in Section 3. The main importance is adopted from the Human Gene Database (genecards.org). N = number of DLM; chr = chromosome; Hypo = hypomethylated loci; Hyper = hypermethylated loci. All parameters for these DMG are shown in Table S2. (**B**) Selected DMG with more than 15 loci in one annotated gene.

(A)			
**Gene**	CpG Probes	Adj. *p*-Value (Min–Max)	Importance
*XRCC5* (N = 2: chr 2)	**Hypo:** cg23433242, cg01633232	5.62 × 10^−3^, 2.79 × 10^−3^	DNA repair–non homologous end joining (NHEJ)
*NR4A2* (N = 9: chr 2)	**Hyper:** cg14617996, cg11209121, cg11358945, cg00194126, cg03339537, cg06101180, cg14811105, cg20570611, cg13500877	4.61 × 10^−3^ – 9.60 × 10^−3^	Autoimmune diseases, neurodegenerative diseases
*CDK2AP1* (N = 9: chr 12)	**Hyper:** cg10289269, cg05760918, cg01660796, cg01247747, cg02017926, cg25326086, cg08696931, cg25630910, cg10411075	7.40 × 10^−4^ – 6.28 × 10^−3^	Reduction in cell proliferation, Cell cycle termination
(**B**)			
**Gene**	**CpG Probes**	**Adj. *p*-Value (Min–Max)**	**Importance**
*RPTOR* (N = 20: chr 17)	**Hypo:** cg12088417, cg17614805, cg27210166, cg00545580 **Hyper:** cg12654199, cg22386583, cg13549638, cg07856428, cg22091236, cg15230985, cg27457201, cg02675920, cg22652378, cg08129331, cg13159505, cg22255288, cg15781195, cg14343513, cg07702089, cg05098037	7.05 × 10^−4^ – 9.15 × 10^−3^	RPTOR encodes part of a signaling pathway regulating cell growth which responds to nutrient and insulin levels.
*COL23A1* (N = 17: chr 5)	**Hypo:** cg06485308, cg02119888 **Hyper:** cg08862479, cg17649857, cg24705158, cg06882830, cg04686319, cg18816996, cg21871330, cg21768539, cg25153741, cg24830898, cg05206633, cg08684511, cg26994283, cg24527008, cg00561180	1.16 × 10^−3^ – 9.08 × 10^−3^	COL23A1 encodes a transmembrane nonfibrillar collagen. This kind of collagen has a single pass hydrophobic transmembrane domain.
*KCNQ1* (N = 16: chr 11)	**Hypo:** cg21752270, cg00243281, cg24492694, cg06827779 **Hyper:** cg23762359, cg05472575, cg12141659, cg11887188, cg16465939, cg08741300, cg11700071, cg21130221, cg05438727, cg05993525, cg07618453, cg06485603	4.62 × 10^−3^ – 9.5 × 10^−3^	This gene encodes a voltage-gated potassium channel required for the repolarization phase of the cardiac action potential.
*PTPRN2* (N = 16, chr 17)	**Hypo:** cg04694534, cg06679384, cg13218485, cg01726608, cg12138780, cg18395809, cg16486501, cg06649856, cg15272173, cg04799270 **Hyper:** cg09656639, cg26208815, cg17291423, cg23634928, cg04007350, cg02704570	2.35 × 10^−3^ – 7.75 × 10^−3^	Protein tyrosine phosphatase, receptor type N2 encodes a major islet autoantigen in type-1 diabetes. PTPRN2 plays an important role in the epigenetic regulation of metabolic diseases and cancers.

Abbreviations: *XRCC5* (X-Ray Repair Cross-Complementing 5), *NR4A2* (Nuclear Receptor Subfamily 4 Group A Member 2), *CDK2AP1* (Cyclin-Dependent Kinase 2-Associated Protein 1), *RPTOR* (regulatory-associated protein of MTOR complex 1), *COL23A1* (Collagen Type-XXIII Alpha 1 Chain), *KCNQ1* (potassium voltage-gated channel subfamily Q member 1), *PTPRN2* (Protein Tyrosine Phosphatase Receptor Type N2).

## Supplementary Materials

The following supporting information can be downloaded at: https://www.mdpi.com/1422-0067/23/3/1666/s1.

## Abbreviations

3′UTR	3′ Untranslated region
B[a]P	Benzo(a)pyrene
BCD	Bisulfite-converted DNA
BMI	Body mass index
CB	Ceske Budejovice
CpG	Cytosine-phosphate-Guanine dinucleotide
DML	Differentially methylated loci
DMG	Differentially methylated groups of loci
EDTA	Ethylenediaminetetraacetic acid
gDNA	Genomic DNA
EWAS	Epigenome-wide association study
FDR	False discovery rate
KEGG	Kyoto Encyclopedia of Genes and Genomes
NHEJ	Non-homologous end joining
PCA	Principal component analysis
PM	Particulate matter
PM2.5	Particulate matter less than 2.5 µm in diameter
SNPs	Single-nucleotide polymorphisms
